# Preparation and Characterization of Responsive Cellulose-Based Gel Microspheres for Enhanced Oil Recovery

**DOI:** 10.3390/gels10080532

**Published:** 2024-08-13

**Authors:** Peng Yin, Fang Shi, Mingjian Luo, Jingchun Wu, Bo Zhao, Chunlong Zhang, Yi Shen, Yanbing Chen

**Affiliations:** 1 College of Chemistry and Chemical Engineering, Northeast Petroleum University, Daqing 163318, China; yinpeng202404@163.com; 2 Daqing Oilfield Company Ltd Natural Gas Sub-Company, Daqing 163000, China; 3 Key Laboratory for EOR Technology (Ministry of Education), Northeast Petroleum University, Daqing 163318, China; sfang1916@163.com (F.S.); kongxiangting2020@163.com (Y.S.); 4 Daqing Oil Field Co., Ltd., No. 6 Oil Production Plant, Daqing 163000, China; zhaobo_0307@163.com; 5 Daqing Yongzhu Petroleum Technology Development Co., Ltd., Daqing 163000, China; 6 Shenyang Oil Production Plant of Liaohe Oilfield, Shenyang 110000, China; hwjtbshgzz1916@163.com

**Keywords:** cellulose-based gel microspheres, enhanced oil recovery, after ASP flooding, alkaline responsive type

## Abstract

As an important means to enhance oil recovery, ternary composite flooding (ASP flooding for short) technology has achieved remarkable results in Daqing Oilfield. Alkalis, surfactants and polymers are mixed in specific proportions and injected into the reservoir to give full play to the synergistic effect of each component, which can effectively enhance the fluidity of crude oil and greatly improve the oil recovery. At present, the technology for further improving oil recovery after ternary composite flooding is not mature and belongs to the stage of technical exploration. The presence of alkaline substances significantly alters the reservoir’s physical properties and causes considerable corrosion to the equipment used in its development. This is detrimental to both the environment and production. Therefore, it is necessary to develop green displacement control agents. In the reservoir environment post-ASP flooding, 2-(methylamino)ethyl methacrylate and glycidyl methacrylate were chosen as monomers to synthesize a polymer responsive to alkali, and then grafted with cellulose nanocrystals to form microspheres of alkali-resistant swelling hydrogel. Cellulose nanocrystals (CNCs) modified with functional groups and other materials were utilized to fabricate hydrogel microspheres. The product’s structure was characterized and validated using Fourier transform infrared spectroscopy and X-ray diffraction. The infrared spectrum revealed characteristic absorption peaks of CNCs at 1165 cm^−1^, 1577 cm^−1^, 1746 cm^−1^, and 3342 cm^−1^. The diffraction spectrum corroborated the findings of the infrared analysis, indicating that the functional modification occurred on the CNC surface. After evaluating the swelling and erosion resistance of the hydrogel microspheres under various alkaline conditions, the optimal particle size for compatibility with the target reservoir was determined to be 6 μm. The potential of cellulose-based gel microspheres to enhance oil recovery was assessed through the evaluation of Zeta potential and laboratory physical simulations of oil displacement. The study revealed that the absolute value of the Zeta potential for gel microspheres exceeds 30 in an alkaline environment with pH values ranging from 7 to 14, exhibiting a phenomenon where stronger alkalinity correlates with a greater absolute value of Zeta potential. The dispersion stability spans from good to excellent. The laboratory oil displacement simulation experiment was conducted using a cellulose-based gel microsphere system following weak alkali ASP flooding within the pH value range from 7 to 10. The experimental interventions yielded recovery rates of 2.98%, 3.20%, 3.31%, and 3.38%, respectively. The study indicates that cellulose-based gel microspheres exhibit good adaptability in alkaline reservoirs. This research offers a theoretical foundation and experimental approaches to enhance oil recovery techniques post-ASP flooding.

## 1. Introduction

Hydrogel microspheres, characterized by high water content and adjustable mechanical properties, are extensively utilized in biomedicine for applications such as biological culture, biosensing, and drug delivery. During the migration of hydrogel microspheres, a shear thinning reaction occurs, leading to improved injectability, followed by the re-establishment of a viscous state post-migration. Hydrogel microspheres with varying compositions, structures, and particle sizes can be utilized to construct composite materials with distinct functional properties [1,2,3]. Conventional hydrogels, being large-scale block structures typically exceeding the millimeter level, fall short of meeting the demands of smaller-scale research and applications. Consequently, the development of nanoscale and microscale hydrogel microspheres is highly significant. The application of hydrogels in oilfield stimulation primarily focuses on two aspects: water shutoff and profile control. By injecting hydrogels, profile control and displacement can be achieved, enhancing reservoir performance and preventing further water infiltration. The application of hydrogels can enhance oil recovery [4,5,6]. The injection of hydrogels alters the flow dynamics of oil and water within the formation, facilitating their separation. Additionally, hydrogels can serve as carriers to deliver other stimulation agents or catalysts into the formation, thereby further improving oil recovery from the reservoir. As a unique polymer material, hydrogels have broad application prospects in oilfield development [7,8,9]. By continuously optimizing their properties and application technologies, hydrogels are expected to play an increasingly significant role in enhancing crude oil production, reducing water channeling, and improving recovery rates. Looking ahead, as oilfield development technology advances and hydrogel research deepens, the application of hydrogels in oilfields is expected to become more extensive and profound. Hydrogels are three-dimensional networks of heterogeneous mixtures. They feature hydrophilic groups and distinctive swelling characteristics. Hydrogels can entrap numerous water molecules within their robust three-dimensional networks via hydrogen bonding, endowing them with strong water retention capabilities. Hydrogels are classified into natural and synthetic polymers based on their origins. Natural polymer hydrogels are characterized by their excellent biocompatibility, extensive sources, and affordability. Natural hydrogels are plentiful, yet relatively unstable. Synthetic polymer hydrogels feature optimized structures for enhanced stability. Currently, the structural designs of hydrogels are diverse, manifesting in various forms. These primarily involve electrostatic adsorption, ionic crosslinking, van der Waals forces, and hydrophobic association. In the presence of porous reservoir rocks, hydrogels experience significant mechanical shear forces during migration, potentially cleaving long-chain polymers into shorter ones. Compared to hydrogels prepared by physical methods, chemical hydrogels contain covalent bonds, forming permanent structures that are less susceptible to external influences. As research into hydrogels progresses, the design of their functionality is shifting from static to dynamic requirements. Physicochemical properties. In recent years, numerous theories and methods have been proposed to enhance the strength of hydrogels, primarily in the following areas [10]: (1) enhancing physical crosslinking between molecules by forming ordered crystals and through molecular chain entanglement; (2) enhancing the uniformity and external impact resistance of the network structure by adjusting monomers and crosslinking agents or optimizing structural design; (3) boosting the mechanical strength of gel materials by creating asymmetric network structures, such as dual and semi-interpenetrating network hydrogels. 

Recently, the use of nanomaterials in oil and gas engineering has been on the rise, with research into nano oil displacement agents to enhance oil recovery being a key area of interest [11,12,13,14,15]. Cellulose nanocrystals (CNCs), as the most promising green alternative to chemical flooding, were designated as a green oil displacement agent by an international association in 2016 [16]. CNC materials, largely sourced from wood via controlled acid hydrolysis, ultimately form rod-shaped particles, making them highly environmentally friendly and capable of being mass-produced [17,18]. The abundance of hydroxyl groups on the CNC surface offers readily accessible reactive sites for functionalization. Additionally, CNC, as a one-dimensional nano-reinforced material, is valued for its superior surface and mechanical properties, which have garnered extensive scientific research interest. Compared to current oil displacement agents, cellulose nanocrystals offer significant environmental benefits. However, the strong hydrophilicity and high specific surface area of CNCs lead to agglomeration, making them challenging for use in organic media. Therefore, surface modification of CNCs is crucial for developing an effective green oil displacement agent. Nano cellulose, not only a natural polymer but also ubiquitous in our surroundings, can be transformed into nano-sized particles, endowing it with superior properties like strong plasticity and high reliability, positioning it as a potential competitor to partially hydrolyzed polyacrylamide (HPAM) in oil displacement. Professor Wei Bing and other researchers [19,20,21] are considering its application in oil displacement agents, combining it with other ASP flooding components. This approach, when compared to conventional ASP flooding, can notably enhance oil recovery. 

This study aims to develop CNC-functionalized gel microspheres to further enhance oil recovery post-ASP flooding. This technology features size controllability, environmental friendliness, and alkali resistance across a broad pH value range. Extensive testing confirms its substantial potential in indoor physical simulations for enhancing oil recovery.

## 2. Results and Discussion

### 2.1. Infrared Spectroscopy Characterization

**Figure 1:**
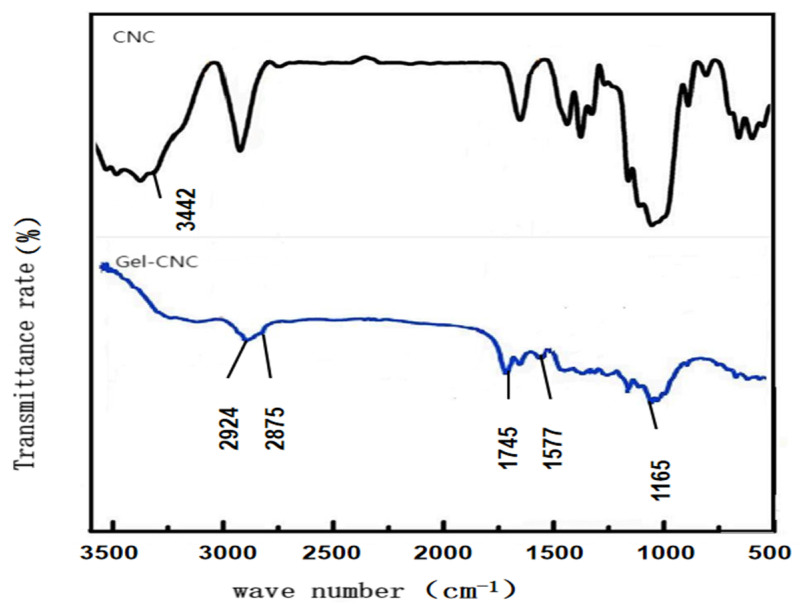
Graph of infrared spectroscopy characterization.

As shown in Figure 1, the initial state of cellulose nanocrystal CNC exhibits a broad and strong characteristic peak at 3342 cm^−1^, which corresponds to the stretching vibration peak of its surface hydroxyl -OH group. Compared with the post reaction, the spectrum of polymethacrylic acid hydrogel microspheres showed new characteristic peaks at 2924 cm^−1^ and 2876 cm^−1^, respectively corresponding to the stretching vibration peaks of methyl-CH_3_ and methylene-CH_2_ groups. The absorption bands observed at 1746 cm^−1^ and 1165 cm^−1^ can be attributed to the characteristic stretching vibrations of the carbonyl C=O and CF_3_ groups in the ester moiety. Another prominent peak near 1577 cm^−1^ confirmed the existence of C-N stretching vibration. In addition, at 3442 cm^−1^ of the hydrogel microspheres, it was observed that the stretching vibration peak of the corresponding hydroxyl group was weaker than that of the original CNC, and all the characteristic absorption peaks appeared in the corresponding spectrogram [22], indicating that the hydrogel microspheres were successfully prepared.

### 2.2. Diffraction Spectral Analysis

**Figure 2:**
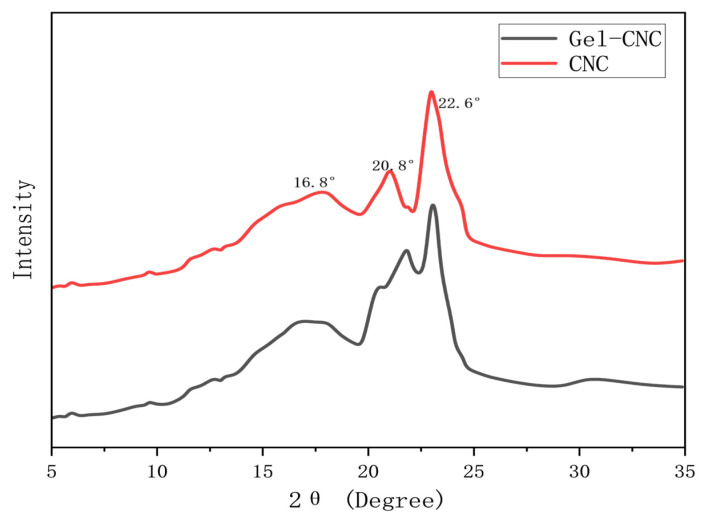
Graph of comparison of diffraction spectra.

The diffraction spectra of cellulose nanocrystals and hydrogel microspheres were compared and analyzed, and the diffraction spectra were compared (XRD) [23]. The X-ray diffraction crystal structures of the original CNC and poly(dimethylaminoethyl methacrylate)–g-CNC-g-PHFBA are presented in Figure 2. The XRD pattern of the original CNC shows three characteristic diffraction peaks at 2 θ = 16.8°, 20.8°, and 22.6°, corresponding to the (110), (102), and (200) crystal planes, respectively.

In addition, the corresponding peak of CNC did not show significant changes after grafting the triblock copolymer, indicating that the modification process only occurred on the surface of CNC and did not disrupt its crystalline structure, which also confirmed the conclusion drawn from infrared spectroscopy.

### 2.3. Laser Particle Size Distribution

**Figure 3:**
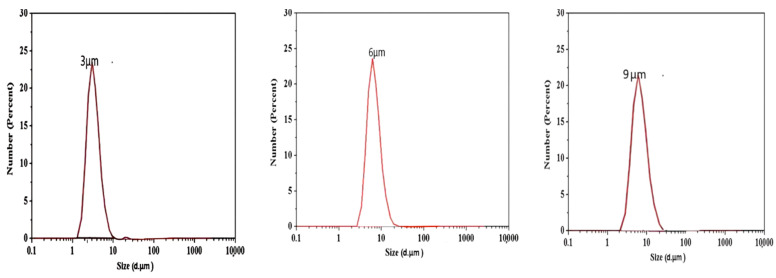
Particle size distribution of hydrogel microspheres.

The three groups of prepared hydrogel microspheres were dispersed in a certain proportion of water. Ultrasonic dispersion was conducted, and the samples were placed in an optical particle size analyzer. Ten statistical calculations for each sample group were performed. The measured particle size distribution diagram is shown in Figure 3. The median diameter of the hydrogel microspheres measured by analyzing three groups of samples was about 3 μm, 6 μm and 9 μm. The particle size distribution chart shows a single peak, indicating relatively reliable data.

### 2.4. Expansion Rate Test

The hydrogel microspheres with the mass concentration of 2 wt% and the median particle size of 3, 6 and 9 μm were immersed in the injection water provided on-site, and the particle size changes after different immersion times were measured by laser particle size analyzer. Then, by adjusting the pH value of the aqueous dispersed system, the dynamic curve of the expansion rate of the hydrogel microspheres changing with the pH value was determined.

**Figure 4:**
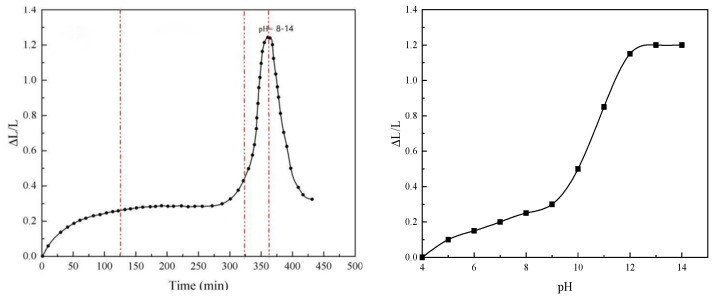
Curve of the variation law of the expansion rate of hydrogel microspheres of 3 to 6 μm.

**Figure 5:**
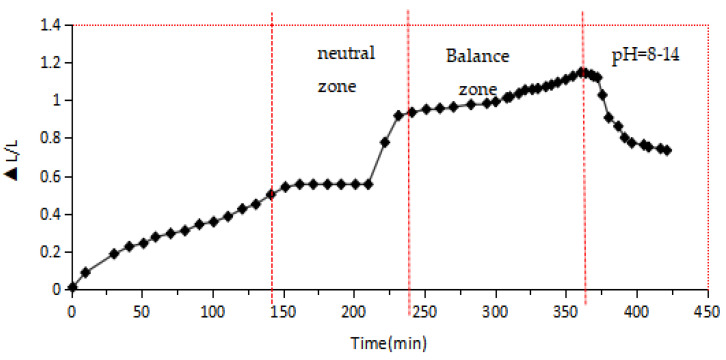
Curve of the variation law of the expansion rate of the 9 μm hydrogel microspheres.

As shown in Figure 4 and Figure 5, in neutral dispersion water, the size of the hydrogel microspheres gradually increased with time, and the expansion rate approached stability at 0.2~0.5 times, entering the equilibrium zone. By adjusting the pH value of the dispersion, we continued to evaluate the size change of the hydrogel microspheres. The results show that when the dispersion environment becomes alkaline and the pH value is in the range of 8~12, the size expansion rate of the hydrogel microspheres continues to increase, and the expansion rate reaches about 1~1.2 times. When the pH value is greater than 13, the expansion rate of the hydrogel microspheres does not change, and the stability time of the expansion rate is shortened.

The dynamic test data showed that the hydrogel microspheres had good volume expansion in an alkaline environment, and the stability time was longer. After optimizing indoor experimental parameters and increasing the application ratio of cellulose nanocrystals and glucose, the expansion stability time can reach more than 3 months.

### 2.5. Flow Performance and Erosion Resistance Test

The flow performance and erosion resistance of hydrogel-dispersed systems with different particle sizes (3 μm, 6 μm, 9 μm) were tested using Bailey cores. We calculated the drag coefficient, residual drag coefficient, and pressure drop rate. The experimental results are shown in Table 1.

Comparing the experimental data of Scheme I and Scheme II, it can be seen that in the stage of injecting hydrogel microspheres, since the hydrogel microspheres in Scheme B are not fully expanded, the pressure at the end of the injection phase is generally lower than that at the end of Scheme A. However, by overall comprehensive comparison, the results of subsequent water drive experiments and scouring resistance experiments after soaking well and waiting for gel expansion are almost the same, with no obvious difference, indicating that the expansion time of hydrogel microspheres has no impact on injection performance.

**Figure 6:**
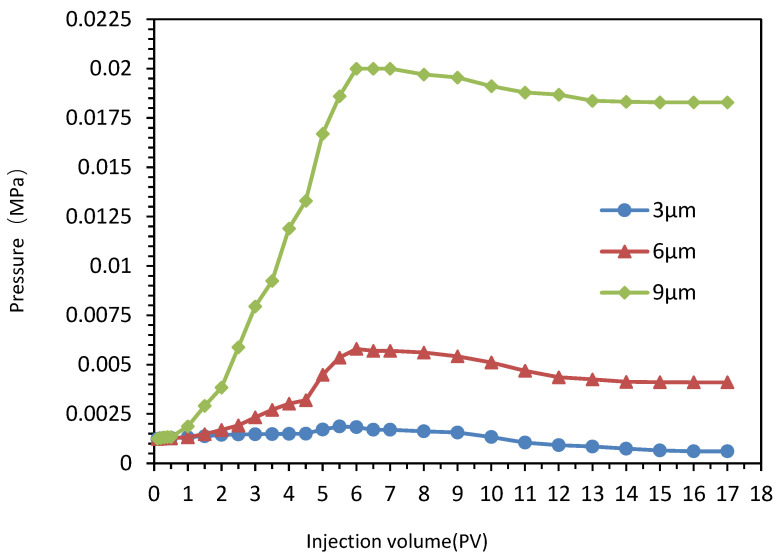
Injection performance curve for hydrogel microspheres with different particle sizes.

As shown in Figure 6, comparing the resistance coefficient and residual resistance coefficient of three particle size hydrogel microsphere dispersed systems, it can be concluded that when injecting hydrogel microsphere dispersed system, because the hydrogel microsphere blocks the core channel, the larger the particle size is, the greater the pressure rise amplitude is, and the greater the resistance coefficient is.

During the subsequent water drive, the injected hydrogel microspheres blocked the pores with high permeability in the rock core, leading to the injection pressure still rising gradually. At the same time, the hydrogel microspheres collided and cemented with each other to become more compact, causing the injection pressure to rise further. When the pressure rises to a certain extent, the pores blocked by the hydrogel microspheres are broken through. At this time, the injection pressure decreases slightly, and the hydrogel microspheres still have large residual resistance.

The 9 μm hydrogel microsphere is almost blocked in the core due to its large particle size, so the injection pressure in the subsequent water drive stage is still rising steadily. To sum up, the 6 μm hydrogel microsphere has excellent injection performance, strong scouring resistance and a good control profile and displacement ability.

### 2.6. Zeta Potential 

**Figure 7:**
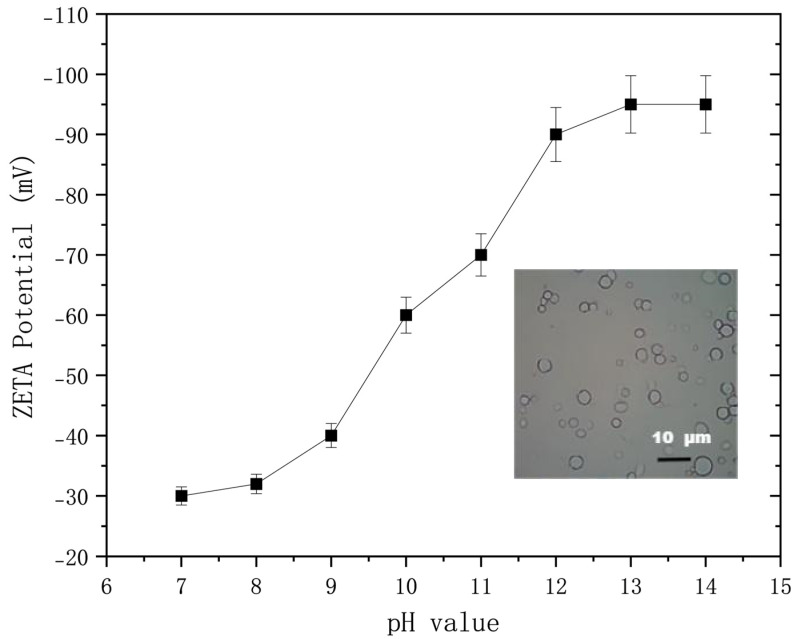
Measured curve of the variation in Zeta potential with pH value.

The significance of Zeta potential lies in its numerical correlation with the stability of colloidal dispersion. The larger the absolute value of the Zeta potential, the more stable the system is. Zeta potential is a measure of the strength of mutual repulsion or attraction between particles. The smaller the molecule or dispersed particle, the higher the absolute value of Zeta potential, and the more stable the system, that is, a dispersed system or dispersion can resist aggregation. On the contrary, the lower the Zeta potential, the more dispersed the system is and the more likely it is to condense or coalesce. Testing the Zeta potential of 2% hydrogel microspheres under different pH values, the size of the gel microspheres determined by the Zeta potential was 6 μm. The experimental results are shown in Figure 7. As the pH value increases, the absolute value of Zeta potential significantly increases. When the pH value is 7–10, the absolute value of Zeta potential is between 32 and 62. When the pH value is greater than 10, the absolute value of Zeta potent7ial is greater than 62, indicating excellent dispersion stability of the composite system. The experimental data showed that the microbial metabolite hydrogel microsphere composite system was relatively stable in an alkaline environment.

### 2.7. Oil Displacement Simulation Experiment

**Figure 8:**
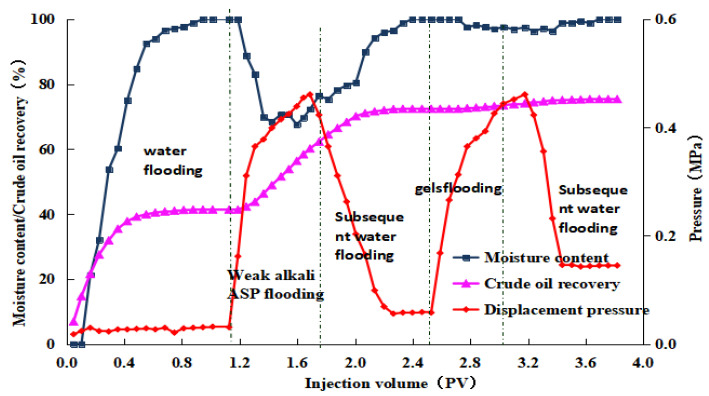
Dynamic curve of gel microdisplacement (pH value = 7).

**Figure 9:**
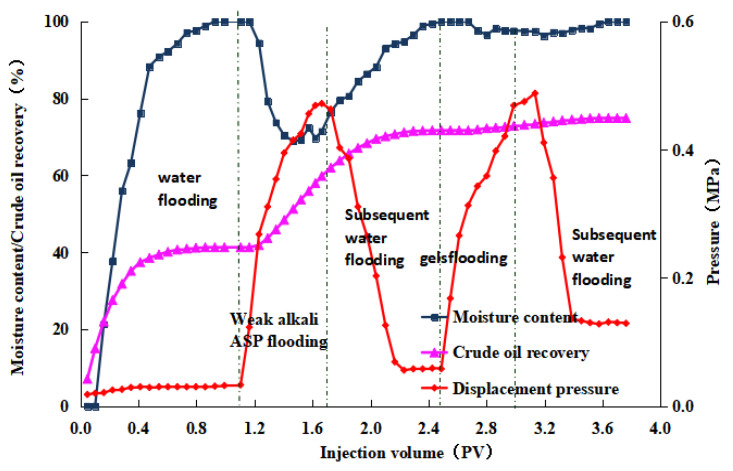
Dynamic curve of gel microdisplacement (pH value = 8).

**Figure 10:**
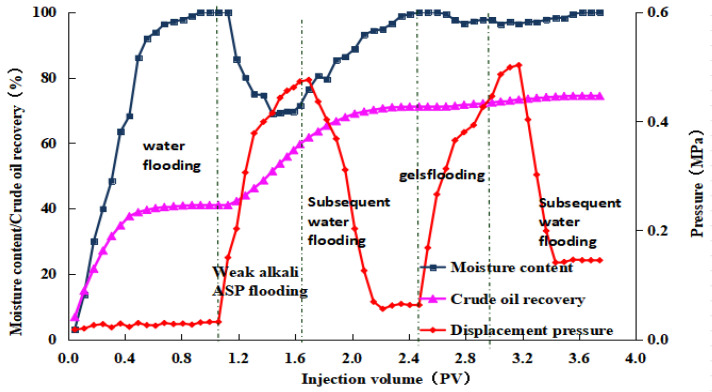
Dynamic curve of gel microdisplacement (pH value = 9).

**Figure 11:**
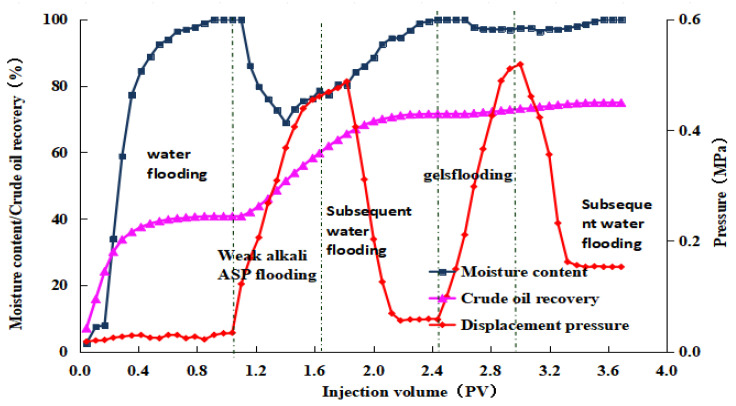
Dynamic curve of gel microdisplacement (pH value = 10).

After the weak alkaline ternary system displacement was complete, we injected a 0.5 PV microbial composite system with varying pH values, followed by a 7-day soak. In the subsequent waterflooding phase, displacement ended when the produced fluid’s water content reached 98%. The post-treated produced fluid was then left to settle to ensure thorough oil–water separation. The oil volume was measured, and the post-treatment recovery rates were calculated to be 2.98%, 3.20%, 3.31%, and 3.38%, respectively. The experimental results for the four groups are displayed in Figure 8, Figure 9, Figure 10 and Figure 11. The study indicates that the microbial composite system exhibits good adaptability in alkaline environments.

## 3. Conclusions

Cellulose-based hydrogel microspheres with three particle sizes were synthesized by self-assembly with functional modified cellulose nanocrystals. The hydrogel microspheres have the characteristics of alkali swelling resistance. The structural characterization was carried out by Fourier transform infrared spectroscopy and X-ray diffraction spectroscopy, confirming the successful preparation of the target product. Through a series of performance evaluations, the research showed that the hydrogel microspheres have better erosion resistance under alkaline conditions. Based on the experimental evaluation of fluidity and erosion resistance, the size of hydrogel microspheres compatible with the target reservoir was determined. The Zeta potential had an absolute value greater than 30 within the pH range of 7–12, indicating good dispersion stability. This study provides a new method for further improving crude oil recovery after ternary composite flooding, which has certain practical value. The laboratory oil displacement simulation experiment was conducted using a cellulose-based gel microsphere system (2 wt%) following weak alkali ASP flooding within the pH value range from 7 to 10. The experimental interventions yielded recovery rates of 2.98%, 3.20%, 3.31%, and 3.38%, respectively. The study indicates that cellulose-based gel microspheres exhibit good adaptability in alkaline reservoirs. Other researchers have also reported similar gel materials, and the research conclusion has reached consensus [24]. This research offers a theoretical foundation and experimental approaches to enhance oil recovery techniques post-ASP flooding.

## 4. Materials and Methods

### 4.1. Experimental Materials

The materials and instruments used in the synthesis and characterization experiments of cellulose-based gel microspheres are shown in Table 2 and Table 3.

### 4.2. Functional Modification Methods for Cellulose Nanocrystals

**Figure 12:**
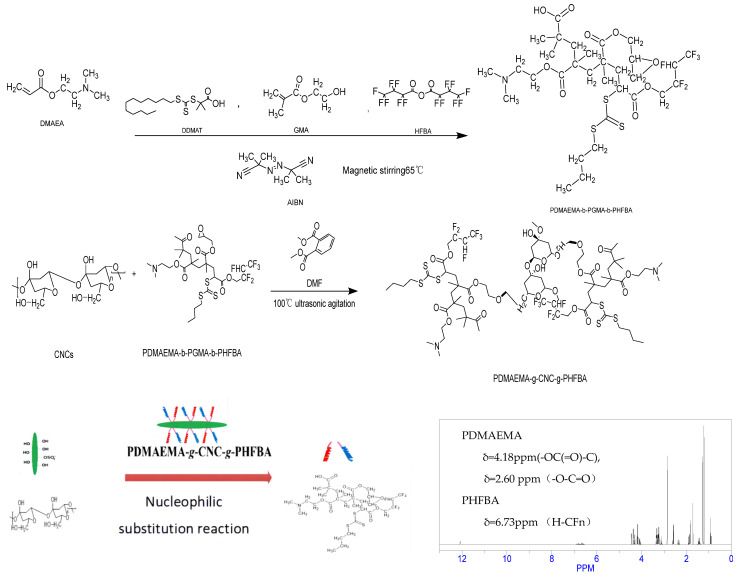
Flow chart of hydrogel microsphere preparation.

Cellulose nanocrystals (CNCs) exhibit agglomeration effects when dispersed in water and require modification in practical applications. The main methods for modifying cellulose nanocrystals are supramolecular assembly and graft polymerization. A CNC modified by responsive functional group grafting exhibited swelling and strong mechanical properties [25,26]. PDMAEMA-b-PGMA-b-PHFBA was used as a basic responsive block copolymer. The preparation process is shown in Figure 12. AIBN (9.0 × 10^−4^ mol), DDMAT (1.1 × 10^−3^ mol), and DMAEMA (5.6 × 10^−2^ mol) were mixed in 1,4-dioxane, and the mixture was stirred magnetically at 65 °C for 10 h under argon protection. Following that, GMA (1.5 × 10^−2^ mol) was mixed in 1,4-dioxane, and the mixture was continuously stirred magnetically at 65 °C for 8 h under argon protection. HFBA (8.1 × 10^−3^ mol) was mixed in 1,4-dioxane, then the mixture was stirred magnetically at 65 °C for 8 h under argon protection. The product was purified in n-hexane using an ice water bath, then dried in a vacuum oven. The alkali-responsive block copolymer PDMAEMA-b-PGMA-b-PHFBA was obtained. In recent years, Professor Jiang Feng’s team at the University of British Columbia has proposed a method of saccharification to prepare hydrogel. This method can effectively solve the performance limitation of hydrogel caused by high free water content. The saccharification method is used to increase the hydrogen bond and intermolecular force of the hydrogel network by adding glucose, which can convert part of the free water of the hydrogel into bound water. Therefore, saccharification endows hydrogels with excellent environmental adaptability. Based on the above reasons, this study improves the interfacial activity and mechanical strength of hydrogels by adding one-dimensional nano reinforced materials cellulose nanocrystals (CNCs) and glucose, and constructs the supramolecular structure of CNC hydrogels [27]. Therefore, glucose is added in the preparation of cellulose-based hydrogel microspheres for assisted dispersion.

### 4.3. Synthesis of Hydrogel Microspheres

As shown in Figure 1, hydrogel microspheres were synthesized by the grafting method. The carboxyl groups of copolymers interact with the hydroxyl groups of cellulose nanocrystals to form ester bonds for crosslinking. The hydrogel microspheres were formed by ultrasonic dispersion. PDMAEMA-b-PGMA-b-PHFBA (3 g) and CNCs (1 g) were ultrasonically dispersed in DMF (40.0 mL) in three parallel aliquots. Next, 1 mL of TEA and glucose (molar ratio 1:1) was added. Magnetic stirring was performed at 100 °C for 24 h under an argon atmosphere. The pH value of the reaction environment was controlled at 10. After the reaction, the modified CNCs were collected by centrifugation at 150 rpm, 400 rpm and 1200 rpm, purified by washing with THF three times, and then dried under vacuum. The molecular weight was measured using the Ubbelohde viscometer counting method [28]. The number-average molecular mass of PDMAEMA-bPGMA-b-PHFBA was 2.3 × 10^4^ g/mol.

### 4.4. Structural Characterization of Hydrogel Microspheres

The structure of cellulose-based nanohydrogel microspheres was characterized by Fourier transform infrared spectroscopy, X-ray diffraction spectroscopy and laser particle size analysis. Infrared spectroscopy analyzers primarily characterize molecular structures and compare the changes in molecular bonds before and after reactions. Infrared light, with wavelengths ranging from 2.5 to 25 μm (or from 4800 to 400 cm^−1^), can be absorbed, transmitted, reflected, and scattered, or induce fluorescence (also known as the Raman effect) when interacting with a sample. Upon absorbing mid-infrared light, molecules undergo vibrational and rotational changes, creating an infrared absorption spectrum with a redispersion system of 2 cm^−1^ and a scanning range from 400 to 4000 cm^−1^. This study employed the coating method for sample testing, involving the application or coating of liquid samples onto salt or window slides to form liquid films for infrared spectral analysis. The X-ray diffraction spectrometer was employed to determine crystal structures, with crystals serving as gratings for X-rays. The coherent scattering produced by tested materials causes light interference, which in turn amplifies or diminishes the intensity of the scattered X-rays. This enables the analysis of structural characteristics before and after modifications, corroborating the findings from infrared spectroscopy. The laser particle size analyzer determines particle size by analyzing the spatial distribution of diffracted and scattered light through Fraunhofer diffraction and Mie scattering theories, unaffected by factors such as temperature fluctuations, medium viscosity, sample density, or surface conditions. Particle size distribution was measured using a Malvern Mastersizer 2000 laser particle size analyzer, with a measurement range from 0.2 to 2000 µm.

### 4.5. Performance Evaluation

#### 4.5.1. Evaluation of the Expansion Rate Measurement

The swelling characteristics of hydrogel microspheres are closely related to the ionic properties in the dispersion environment. In the swollen state, water molecules are subjected to the action of functional groups on the polymer chain and network blocking. In hydrogels, water is bound water, intermediate water in the network and free water outside the network. Under the influence of different external environmental conditions, the water ratio of these three states changes dynamically, which causes the volume of gel to swell or shrink. Hydrogel microspheres with the mass concentration of 2 wt% and the median particle size of 3, 6 and 9 μm were immersed in the injection water provided on-site. A laser particle size analyzer was used to measure the changes in particle size after different soaking times. Then, by adjusting the pH value of the aqueous dispersed system, the dynamic curve of the expansion rate of the hydrogel microspheres changing with the pH value was determined.

#### 4.5.2. Wash-Resistance Experiment

In the reservoir porous media post ASP flooding, assessing the alkali and shear resistance of hydrogel is critical. The resistance and residual resistance coefficients of the hydrogel microspheres are determined via core fluidity experiments, along with evaluating the swelling capacity of the prepared microspheres and their compatibility with the target reservoir cores [29]. To investigate the influence of expansion time on the injectability and scouring resistance of the hydrogel dispersed system, two scenarios were designed for comparative testing. The displacement pressures for the different scenarios were recorded, and the resistance, residual resistance, and pressure drop rates were calculated for comparison. 

Scenario I: Following water flooding, inject the expanded hydrogel system into the core, then perform subsequent water flooding and scouring resistance experiments, recording the pressure at each displacement stage (water flooding pressure P_1_ + hydrogel system pressure P_2_ + subsequent water drive pressure P_3_ + scouring resistance pressure P_4_). Scenario II: After water flooding, inject hydrogel dispersed systems with mass concentrations of 2 wt% and median particle sizes of 3, 6, and 9 μm, respectively. Soak for 7 days to allow the hydrogel to expand, then perform subsequent water flooding and scouring resistance experiments, recording the pressure at each displacement stage (water flooding pressure P_1_ + hydrogel system pressure P_2_ + subsequent water flooding pressure P_3_ + scouring resistance pressure P_4_).

The resistance coefficient (R_f_), is an important indicator that characterizes the injection capacity of displacement agents. Essentially, it is the ratio of water flowability to displacement fluid flowability, and the formula is as follows (1):

(1) $${\;R}_{f}=\frac{\lambda_{w}}{\lambda_{p}}=\frac{k_{w}/\mu_{w}}{k_{p}/\mu_{p}}$$

When ignoring the influence of viscoelasticity, if the length of the porous medium is constant and the flow rates of the displacement system solution and water are constant, using Darcy’s formula, the above equation can be changed to

(2) $$R_{f}=\frac{{\left(\Delta P/\mathrm{LV}\right)}_{s}}{{\left(\Delta P/\mathrm{LV}\right)}_{w}}$$

If the length of the porous medium is constant and the flow rate of the oil displacement system and water is constant, then

(3) $$R_{f}=\frac{{\left(\Delta P\right)}_{p}}{{\left(\Delta P\right)}_{w}}$$

In experiments, the following methods are often used to determine the resistance coefficient (R_f_) and residual resistance coefficient (R_ff_). Record the stable pressure of water flooding, system flooding, and subsequent water flooding. Then, for the same core sample, if the injection rate remains constant under two conditions (before and after the injection system), then:

(4) $$R_{f}=\frac{{\left(\Delta P\right)}_{p}\left(\mathrm{system}\;\mathrm{flooding}\right)}{{\left(\Delta P\right)}_{w}\left(\mathrm{water}\;\mathrm{flooding}\right)}=\frac{P_{2}}{P_{1}}\;R_{\mathrm{ff}}=\frac{{\left(\Delta P\right)}_{w}\left(\mathrm{Subsequent}\;\mathrm{water}\;\mathrm{flooding}\right)}{{\left(\Delta P\right)}_{w}\left(\mathrm{water}\;\mathrm{flooding}\right)}=\frac{P_{3}}{P_{1}}$$

After achieving stable pressure through subsequent water flooding, continue to inject simulated formation water for displacement experiments to simulate the underground scouring environment. Record the pressure changes after displacing a certain volume of simulated formation water to assess the system’s scouring resistance. If the pressure drop is small and can be maintained in a relatively stable state, the system demonstrates strong scouring resistance. Conversely, a continuous or significant decrease in pressure indicates weak scouring resistance. This study characterizes the system’s scouring resistance through the pressure drop rate, with the formula detailed as follows: $\frac{P_{3}-P_{4}}{P_{3}}$.

Among the above categories:

$R_{f}$—Resistance coefficient (dimensionless); $R_{\mathrm{ff}}$—Residual drag coefficient (dimensionless); $\lambda_{w}$—The flow rate of the water passing through the same core; $\lambda_{p}$—The flow rate of the system passing through the same core; $K_{w}$—The permeability of water (×10^−3^ μm^2^); $K_{p}$—The permeability of the injection system (×10^−3^ μm^2^); $\mu_{w}$—Viscosity of water (mPa·s); $\mu_{p}$—Viscosity of the injection system (mPa·s); L—Core length (m); V—Volume flow rate (m^3^/d); ΔP—Pressure drop at both ends of the rock core before and after fluid flow (MPa).

#### 4.5.3. Zeta Potential Analysis

The magnitude of Zeta potential can be used to effectively evaluate the stability of the system. Zeta potential is a measure of the strength of mutual repulsion or attraction between particles. The smaller the molecule or dispersed particle, the higher the absolute value (positive or negative) of Zeta potential, and the more stable the system, that is, the dispersed system or dispersion can resist aggregation. On the contrary, the lower the Zeta potential (positive or negative), the more inclined it is to condense or coalesce, that is, the attractive force exceeds the repulsive force, and the dispersion is destroyed and condenses or coalesces. The stability of the dispersed system was analyzed by measuring the Zeta potential of the hydrogel microsphere system with different mass concentrations. Based on the DLVO theory, the larger the absolute value of Zeta potential, the better the stability of the system. According to Table 4, the Zeta potential corresponds to the colloidal stability of the dispersed system.

#### 4.5.4. The Laboratory Oil Displacement Simulation Experiment 

The experimental temperature was set at 45 °C. The oil used was a simulated oil (9.8 mPa·s) formulated from the crude oil of the oilfield reservoir. The experimental water comprised simulated formation water (6778 mg/L) formulated on-site and the produced water from the oilfield. The oil displacement agent employed a weak alkali ternary system and hydrogel microspheres with a particle size of 6 μm. The experimental core was a natural core measuring 4.5 cm × 4.5 cm × 30 cm with an effective permeability of 600 × 10^−3^ μm^2^. Experimental procedures: (1) The cores were placed in a long core holder with screens at both ends to minimize end effects, and the temperature was set at a constant 45 °C. (2) The core permeability was measured using a gas, followed by evacuating the core for 4 h, saturating it with simulated formation water, and then maintaining it at temperature for 24 h while calculating the core pore volume using a prescribed formula. (3) The ISCO pump was set to constant flow mode at a rate of 0.3 mL/min. After the pressure stabilized, readings were recorded, and the water phase permeability was calculated. Cores with similar permeability were selected for further study. (4) The prepared simulated oil was injected into the core, and after saturation, the quantity of saturated oil was calculated and maintained at temperature for 24 h. (5) Various simulated oil displacement experiments were conducted as per the experimental design, and the fluid output at the outlet was observed. (6) The liquid volume at the outlet was continuously measured, and the experiment was terminated when the water content reached 98%. Experimental protocol: water flooding + weak alkali ternary system + subsequent water flooding (injection parameters provided by the oilfield) + 2% cellulose hydrogel microsphere system (injection volume of 0.5 PV) + continued water flooding until 98% water content, with pH values of 7, 8, 9, and 10 in parallel experiments.

## Figures and Tables

**Table 1:** Experimental results for fluidity and resistance to erosion.

Scheme	Core	Particle Size/μm	Resistance Factor	Residual Resistance Factor	Pressure Reduction Rate (%)
I	A-1	3	1.16	1.32	0.64
	A-2	6	2.52	4.49	0.28
	A-3	9	10.00	15.04	0.09
II	B-1	3	1.09	1.33	0.62
	B-2	6	2.36	4.51	0.28
	B-3	9	9.29	14.96	0.09

**Table 2:** List of specific parameters of main experimental materials.

No.	Name of Chemical Agent	Physical Parameters	Supplier	Purpose
1	Cellulose nanocrystals (CNC ≥ 94%)	Liquid; 200 nm in length and 10 nm in diameter	MACKLIN Biochemical Technology Co., Ltd., Shanghai, China	For synthesis of target gel microspheres
2	2-(methylamino) ethyl methacrylate 3-(DMAEMA, 99%)	Liquid, AR	Merck Group company, Darmstadt, Germany	Preparation of responsive copolymers
3	Glycidyl methacrylate (GMA, 97%)	Solid, AR	MACKLIN Biochemical Technology Co., Ltd., Shanghai, China	Preparation of responsive copolymers
4	Hexafluorobutyl acrylate (HFBA, 97%)	Solid, AR, filtered through neutral aluminum oxide column prior to use	Anpu Experimental Technology Co., Ltd., Shanghai, China	Preparation of responsive copolymers
5	Azobisisobutyronitrile (AIBN, 98%)	Solid, AR	Haiqu Chemical Co., Ltd., Shanghai, China	Polymerization initiator
6	N,N-dimethylformamide (DMF)	Liquid, AR	Century Tongda Chemical Co., Ltd., Jinan, China	For PDMAEMA-b-PGMA-b-PHFBA branch to the surface of CNCs
7	Triethylamine (TEA)	Liquid, AR	Chemical Reagent Co., Ltd., Tianjin, China	For PDMAEMA-b-PGMA-b-PHFBA branch to the surface of CNCs
8	2-(((dodecylthio)carbonothioyl)thio)-2-methylpropanoic acid 3-(DDMAT)	Solid, AR	Merck Group company, Darmstadt, Germany	Raft controlled radical polymerization promoter
9	Tetrahydrofuran (THF)	Liquid, AR	MACKLIN Biochemical Technology Co., Ltd., Shanghai, China	Purification of copolymer
10	Core model	Natural core	Provided by Daqing oilfield	Core flowability testing and oil displacement experiment

**Table 3:** List of specific parameters of main experimental instruments.

No.	Name of Instrument	Model Specifications	Supplier	Purpose
1	Fourier transform infrared spectrometer	Nicolet iS50	Thermo Fisher Scientific Molecular Spectroscopy Company, Shanghai, China	Structural characterization of synthetic products
2	X-ray diffraction spectrometer	TD-5000	Tongda Technology Co., Ltd. Dandong, China	Structural characterization of synthetic products
3	Laser particle size analyzer	Mastersizer 2000 (from 0.2 to 2000 μm)	Pudi Biotechnology Co., Ltd., Shanghai, China	Evaluation of particle size distribution in dispersed systems
4	Zeta potential tester	ZETASIZER NANA ZS (from 3.8 nm to 100 μm)	Sibaiji Instrument System Co., Ltd., Shanghai, China	Dispersion stability evaluation
5	Oil displacement simulation device	Thermostat, core holder, ISCO pump, constant speed and pressure pump, intermediate container	Designed and assembled by the laboratory	Core flowability testing and oil displacement experiment

**Table 4:** Reference table for the colloid stability degree of the dispersed system corresponding to the Zeta potential.

Zeta Potential Absolute Value (mV)	Stability of Dispersed System
<5	Rapid coagulation or condensation stage
10~30	Stability is average
30~40	Stability good
40~60	Stability better
>60	Stability excellent

## Data Availability

The data presented in this study are available in the article.
